# Gene expression pattern of Treg and TCR Vγ subfamily T cells before and after specific immunotherapy in allergic rhinitis

**DOI:** 10.1186/1479-5876-12-24

**Published:** 2014-01-25

**Authors:** Rui Zheng, Xiuli Wu, Xuekun Huang, Yulian Chen, Qintai Yang, Yangqiu Li, Gehua Zhang

**Affiliations:** 1 Department of Otorhinolaryngology-Head and Neck Surgery, The Third Affiliated Hospital, SUN Yat-sen University, Guangzhou 510630, China; 2 Institute of Hematology, Medical College, Jinan University, Guangzhou 510632, China

**Keywords:** Allergic rhinitis, Specific immunotherapy, Foxp3, Treg, γδ T cells

## Abstract

**Background:**

T regulatory cell (Treg) plays a critical role in respiratory allergy and allergen-specific immunotherapy (SIT), and γδ T cells might participate in mediating Treg quantity and/or function in some immunological diseases. To further characterize whether γδ T cells could influence Treg in allergic rhinitis (AR) and SIT, we investigated the expression pattern of Treg’s Foxp3 gene and γδ T cell receptor (TCR) Vγ subfamily genes in peripheral blood mononuclear cells (PBMCs) of AR patients before and after SIT.

**Methods:**

Eighteen AR patients undergoing effective SIT with house dust mite extract for one year were recruited. Visual Analogue Scale (VAS) was applied to evaluate the severity. Immunofluorescence quantification analysis was performed to determine the serum specific IgE (sIgE) content. Real-time PCR was used to detect the expression levels of Foxp3 and TCR Vγ subfamilies. Ten healthy volunteers were recruited as the controls.

**Results:**

Nasal uni-VAS score after SIT was significantly lower than that before SIT, while serum sIgE content was similar before and after SIT. Expression levels of Foxp3 and TCR Vγ subfamilies in AR patients before treatment were significantly lower than those in healthy subjects. Expression levels of VγI and II were similar before and after SIT, while expression levels of Foxp3 and VγIII after SIT were significantly higher than those before. Before SIT, the significant positive correlation was observed between expression levels of Foxp3 and VγI, II, III, while negative correlation was observed between Foxp3, VγIII and VAS. After SIT, the significant positive correlation between expression levels of Foxp3 and VγIII and negative correlation between Foxp3, VγIII and VAS were observed.

**Conclusions:**

Treg and Vγ subfamily T cells were in a dynamic equilibrium in AR patients before and after effective immunotherapy for one year. The early improvement of symptoms following immunotherapy might be independent of the serum sIgE content in AR patients, but associated with the reconstitution of T cell immunity.

## Background

Allergic Rhinitis (AR) is part of the systemic allergic disease, which involves the formation of specific IgE antibodies against innocuous environmental substances. Allergen-specific immunotherapy (SIT) is currently the only therapeutic choice to alter the natural course of AR and has gradually become the first-line treatment, yet its functional mechanism has not been fully elucidated. It has been reported that T regulatory cell (Treg) quantity and/or function were reduced in allergic diseases [1], and SIT could make the quantity rebound and/or the function recover [2]. Previous studies from others [3] and us [4] have found that CD4^-^CD8^-^ γδ T cells predominantly expressed in mucous membrane participate in respiratory allergy. Various functional subsets of γδ T cells could promote or suppress allergic inflammation under different conditions, and clinical effectiveness of SIT was possibly related to the induction of inflammation suppression by the subsets of γδ T cells [5]. It was confirmed in a recent study that γδ T cells could limit Treg quantity and inhibit its function by generating plenty of IL-17, thus promoting autoimmune inflammation [6]. However, the role of γδ T cells in allergic inflammation has not yet been reported. The aim of this study was to investigate the expression pattern of different γδ T cell subfamilies (subsets) as well as their potential correlation with Treg in untreated AR and after early SIT by testing the expression levels of Foxp3 and TCR VγI ~ III genes in AR patients before and after one-year-long effective SIT.

## Methods

### Sample collection

The subjects of the study were 18 patients with moderate to severe persistent AR sensitized to house dust mites (7 males and 11 females; median age: 25 years, range: 6 ~ 38 years). All patients were diagnosed based on ARIA guideline (Allergic Rhinitis and its Impact on Asthma; 2008 update [7]), and received SIT with standardized house dust mite extract (standardized immunotherapy protocol of the University of Copenhagen, Denmark) for 1 year at the Third Affiliated Hospital of Sun Yat-Sen University. The clinical characteristics of the patients were listed on Table 1. The therapy was effective based on the effective criteria [8]. Peripheral blood samples were obtained from the patients at the beginning of their immunotherapy and after one year’s treatment. One month before SIT, all subjects were withheld from oral corticosteroids, antihistamines or immunotherapy. Ten healthy individuals with no symptom of any allergic diseases and negative serum sIgE served as the control group (4 males and 6 females; median age: 27.5 years, range: 16 ~ 40 years). Serums were separated by low-speed centrifugation. The PBMCs were isolated using Ficoll-Hypaque gradient centrifugation method. RNA extraction and cDNA synthesis was performed according to the manufacturer’s instructions [9]. All samples were obtained with written informed consent from the participants, and all of the procedures were conducted according to the guidelines of the Medical Ethics Committee of Health Bureau of China before study initiation.

**Table 1:** Clinical characteristics of AR patients

**Case number**	**Sex**	**Age (years)**	**VAS score before SIT**	**VAS score after SIT**
1	Female	10	8.36	2.87
2	Male	22	7.94	2.37
3	Male	25	7.92	0.46
4	Female	25	7.89	1.74
5	Female	36	8.09	3.82
6	Male	12	7.03	3.54
7	Female	31	7.35	1.47
8	Male	31	7.83	1.72
9	Female	38	6.96	2.13
10	Female	6	7.51	1.68
11	Female	12	7.85	2.26
12	Female	21	7.97	3.68
13	Male	27	8.27	2.54
14	Female	17	8.01	0.37
15	Female	35	7.99	0.96
16	Male	25	7.63	2.44
17	Male	21	7.42	3.06
18	Female	37	7.72	3.18

### Visual analogue scale

The severity of the nasal symptoms of 18 AR patients before and after immunotherapy was assessed according to Visual Analogue Scale (VAS) [10]. The global discomfort caused by AR during the previous week of the test was rated on a 0-10 scale, 0 being no symptom, and 10 being the maximal severity of the symptom.

### Serum specific IgE detection

The contents of serum specific IgE (sIgE) against the house-dust-mite allergen before and after SIT were detected using the automatic system of immunofluorescence quantitative analysis (UniCAP 100E; Pharmacia, Sweden).

### Real-time polymerase chain reaction for Foxp3 and TCR VγI ~ III subfamily genes

Real-time polymerase chain reaction (PCR) was used to determine Foxp3 and TCR VγI ~ III gene expression levels in the PBMCs from samples. Beta 2-microglobulin (β_2_M) gene was used as an endogenous reference. The sequences of primers were listed in Table 2. PCR was performed as previously described [11,12]. In brief, 20 μl PCR reaction mixture containing approximately 1 μL of cDNA, 0.6 mmol/L of each primer, and 10 μl of 2.5× Real Master Mix (Tiangen Biotech (Beijing) Co. Ltd., Beijing, China) was prepared. Amplification was carried out on the CFX96™ Real-Time PCR cycler (Bio-Rad, Hercules, CA, USA) using the cycling conditions as follows: 15 minutes’ initial denaturation at 95°C, 44 cycles consisted of 30 seconds at 95°C; 40 seconds at 60°C; and 2 seconds at 82°C. The melting curve was obtained from 55 to 95°C (0.5°C/s). The 2^(–ΔCT)^ method was used to calculate the relative amount of the genes of interest.

**Table 2:** Sequences of primers used in real-time PCR

**Primers**	**Sequences**
Foxp3-f	5′-CTGACCAAGGCTTCATCTGTG-3′
Foxp3-b	5′-ACTCTGGGAATGTGCTGTTTC-3′
VγI	5′-TACCTACACCAGGAGGGGAAG-3′
VγII	5′-GGCACTGTCAGAAAGGAATC-3′
VγIII	5′-TCGACGCAGCATGGGTAAGAC-3′
Cγ	5′-CATCTGCATCAAGTTGTTTATC-3′
β_2_M-f	5′-TACACTGAATTCCACCCCCAC-3′
β_2_M-b	5′-CACTCAATCCAAATGCGGCA-3′

### Statistical analysis

The Student’s t test or Mann-Whitney U test was performed to compare the means of gene expression levels in different groups. Data were presented as mean ± SD. Pearson correlation or Spearman’s rank correlation analysis was used to estimate the correlations. Statistical analysis was performed using SPSS version 16.0 statistic software package. Differences were considered statistically significant at *P* < 0.05.

## Results

### VAS scores and serum sIgE contents before and after SIT in AR patients

Mean nasal uni-VAS score of 18 AR patients at the beginning of treatment was 7.61 ± 0.34, and after immunotherapy for one year significantly decreased to 2.02 ± 0.98 (t = 20.772, P < 0.001), indicating an alleviation of the nasal hypersensitivity. The mean content of serum sIgE was similar before and after SIT (22.17 ± 8.64 kU/L and 19.72 ± 7.18 kU/L, respectively; Z = 1.051, P = 0.278).

### Expression pattern of Foxp3 and TCR Vγ subfamilies in AR patients and healthy subjects

**Figure 1:**
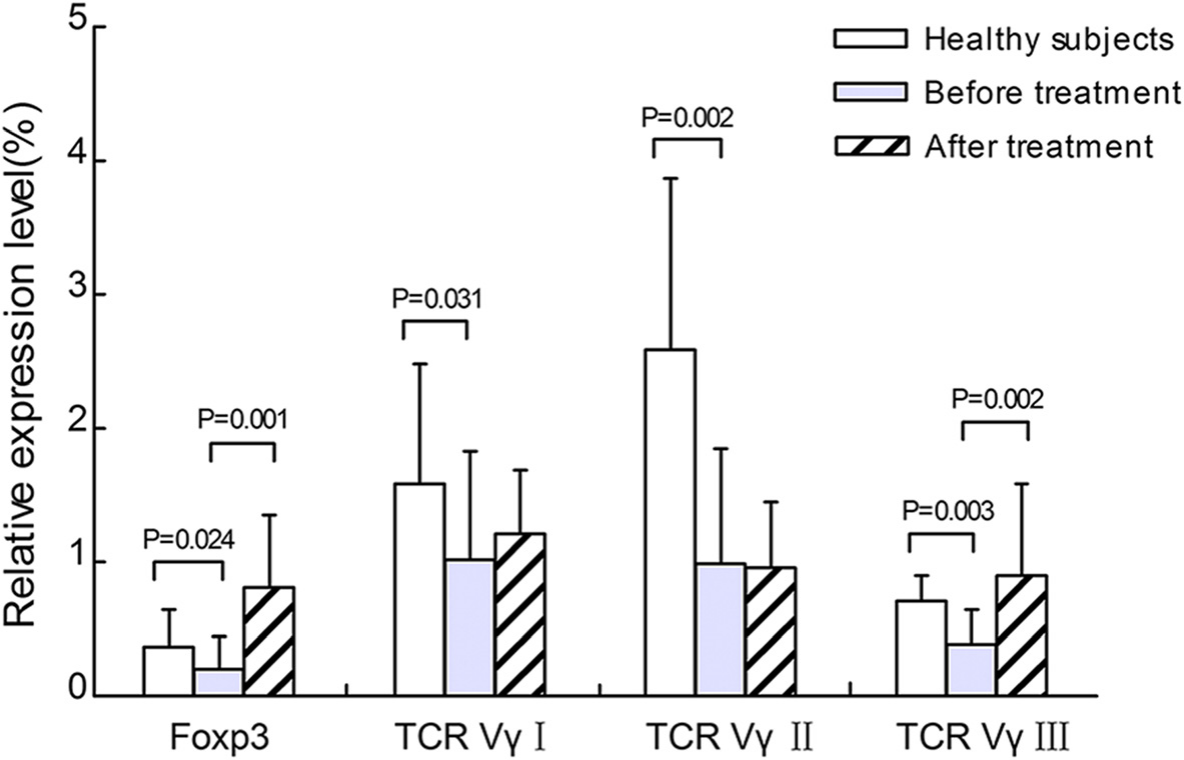
The relative expression levels of Foxp3 and TCR Vγ subfamilies before and after SIT.

The expression pattern of TCR Vγ subfamily genes was VγII > VγI > VγIII in PBMCs from healthy subjects. In contrast, it was VγI > VγII > VγIII in AR patients both before and after SIT. Significantly lower expression levels of Foxp3 (0.20 ± 0.25), VγI (1.02 ± 0.81), VγII (0.99 ± 0.86) and VγIII (0.39 ± 0.26) were observed in untreated AR patients compared with healthy subjects (Foxp3: 0.37 ± 0.28, *Z* = -2.253, *P* = 0.024; VγI: 1.59 ± 0.89, *Z* = -2.158, *P* = 0.031; VγII: 2.59 ± 1.28, *Z* = -3.069, *P* = 0.002; VγIII: 0.71 ± 0.19, *t* = 3.345, *P* = 0.003). After SIT, the expression levels of VγI and VγII in AR patients were 1.21 ± 0.48 and 0.96 ± 0.49, respectively, not significantly different from those before SIT (VγI: *t* = -0.977, *P* = 0.342; VγII: *Z* = -0.501, *P* = 0.616, respectively). However, the expression levels of Foxp3 and VγIII were obviously increased after SIT (Foxp3: 0.81 ± 0.54, *t* = -0.977, *P* = 0.342; VγIII: 0.90 ± 0.69, *Z* = -0.501, *P* = 0.616) (Figure 1).

### Correlation analysis of Foxp3 and TCR Vγ subfamily genes

**Figure 2:**
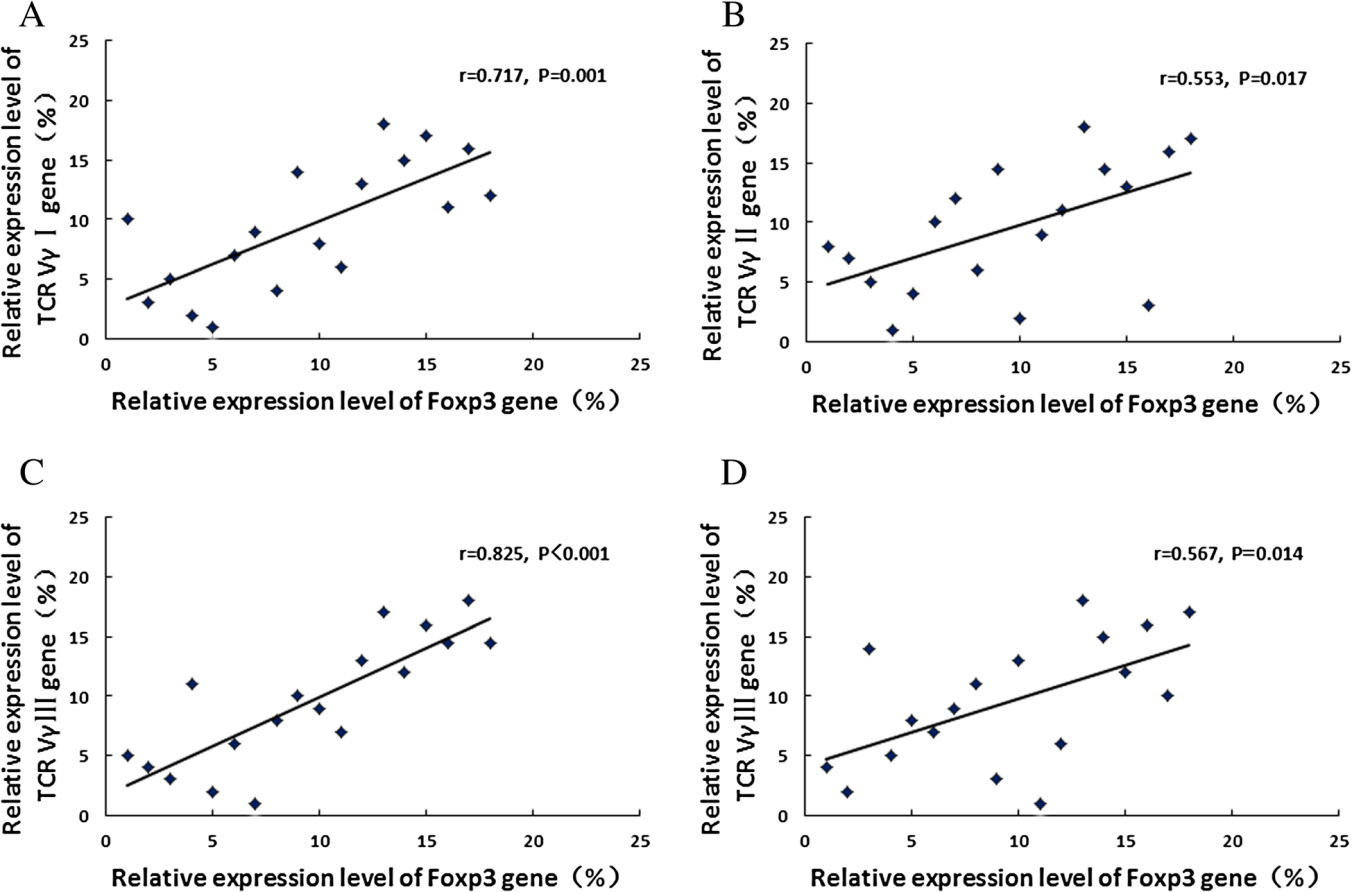
**Correlations between Foxp3 and TCR Vγ gene expression levels before and after SIT. (A)** Foxp3 *vs.* TCR VγI in the before SIT group; **(B)** Foxp3 *vs.* TCR VγII in the before SIT group; **(C)** Foxp3 *vs.* TCR VγIII in the before SIT group; **(D)** Foxp3 *vs.* TCR VγIII in the after SIT group. Significant positive correlations were indicated.

There was no significant correlation between the expression levels of Foxp3 and TCR VγI ~ III genes in healthy subjects (*P* = 0.533, *r* = 0.224; *P* = 0.082, *r* = -0.576; *P* = 0.987, *r* = 0.006, respectively). Significant positive correlation of the expression levels of Foxp3 and TCR VγI ~ III genes was found before SIT (*P* = 0.001, *r* = 0.717; *P* = 0.017, *r* = 0.553; *P* < 0.001, *r* = 0.825, respectively). After SIT, the expression level of Foxp3 had no significant correlation with those of TCR VγI or VγII genes (*P* = 0.932, *r* = 0.022; *P* = 0.367, *r* = 0.226), but was positively related to the expression level of VγIII gene (*P* = 0.014, *r* = 0.567) (Figure 2).

### Correlation analysis of VAS score, Foxp3 and TCR Vγ subfamily genes

**Figure 3:**
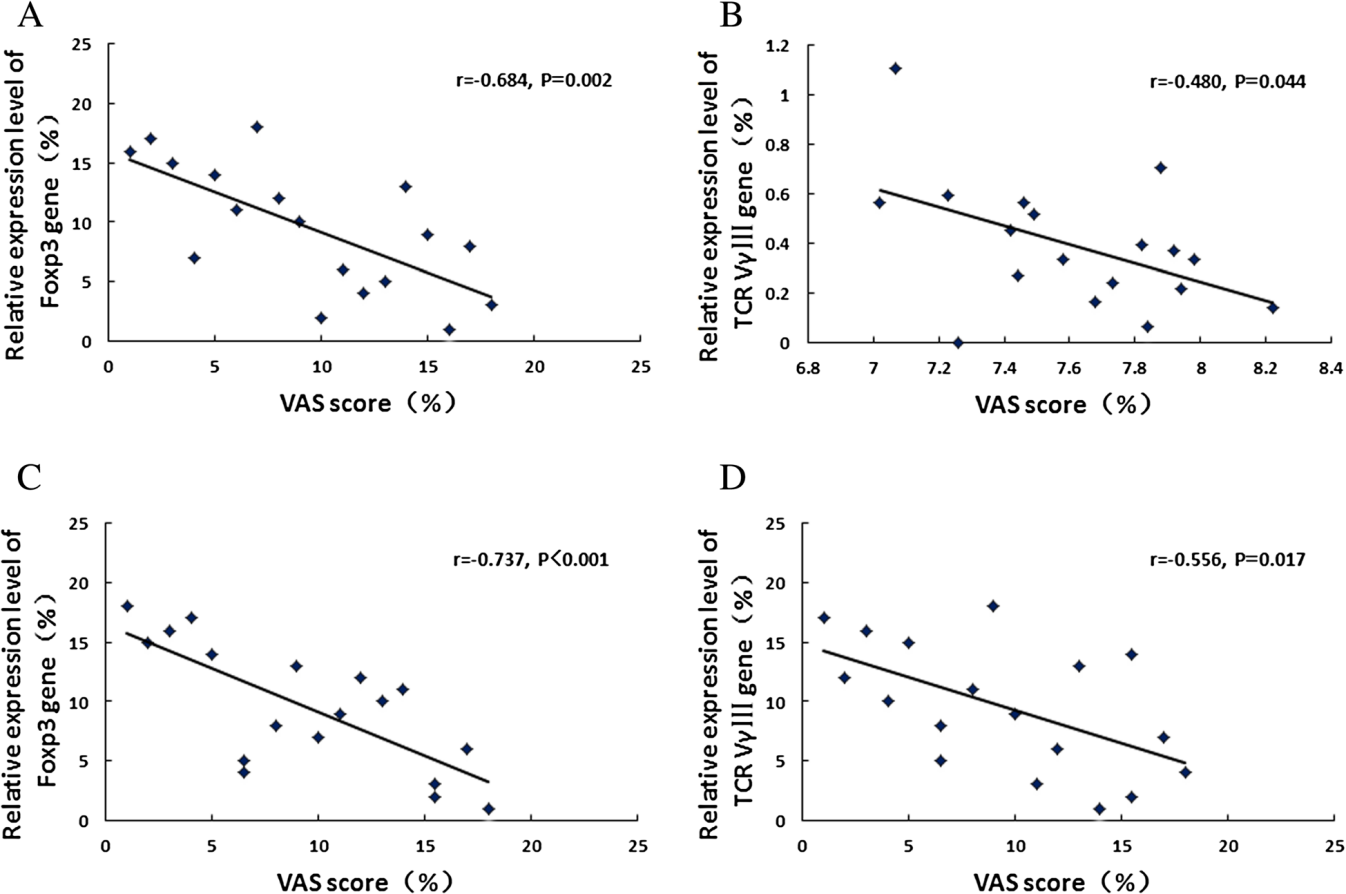
**Correlations between VAS score, Foxp3 and TCR Vγ gene expression levels before and after SIT. (A)** VAS *vs.* Foxp3 in the before SIT group; **(B)** VAS *vs.* TCR VγIII in the before SIT group; **(C)** VAS *vs.* Foxp3 in the after SIT group; **(D)** VAS *vs.* TCR VγIII in the after SIT group. Significant negative correlations were indicated.

In AR patients before SIT, significant negative correlation was observed between the VAS score and the expression level of Foxp3 (*P* = 0.002, *r* = -0.684), and between the VAS score and the expression level of TCR VγIII (*P* = 0.044, *r* = -0.480). After SIT for one year, with the expression level of Foxp3 gene increasing and TCR VγIII decreasing, significant negative correlations remained between the levels of these two genes and the VAS score (*P* < 0.001, *r* = -0.737; *P* = 0.017, *r* = -0.556, respectively) (Figure 3).

### Correlation analysis of Foxp3, TCR Vγ subfamily genes, VAS score and sIgE levels

No significant correlation was shown between the expression levels of Foxp3, TCR Vγ subfamily genes, VAS score and the contents of serum sIgE in AR patients before or after SIT for one year. (Before SIT: *P* = 0.763, *r* = -0.076; *P* = 0.711, *r* = -0.094; *P* = 0.720, *r* = -0.091; *P* = 0.750, *r* = 0.081; *P* = 0.943, *r* = 0.018, respectively. After SIT: *P* = 0.403, *r* = -0.210; *P* = 0.380, *r* = -0.220; *P* = 0.556, *r* = 0.149; *P* = 0.475, *r* = -0.180; *P* = 0.657, *r* = 0.112, respectively).

## Discussion

SIT could restore the body’s immune tolerance and also rectify the body’s immune imbalance, making it the only etiological therapy for allergic diseases at present [13]. Though SIT has been practiced for more than a hundred years, and its safety and effectiveness have been confirmed with the implement of standardized allergen vaccines, it has not been well clarified regarding its role during the course of therapy. AR is a kind of type I allergic disease primarily mediated by IgE. Serum sIgE level is the important clinical criteria to diagnose AR, and is supposed to decline in patients under effective SIT. However, recent studies have shown that serum sIgE level increased but not decreased in the early stage of SIT, and a long therapeutic process was required to observe the reduction in sIgE [14,15]. Similar results were found in our study, which showed that the serum sIgE level after one-year-long SIT was similar to that before SIT. Moreover, there was no significant correlation between sIgE level and VAS score. These data suggest that serum sIgE level has no linear correlation with the improvement of clinical symptoms in the early stage of SIT, thus it cannot serve as an objective criteria to evaluate the effectiveness of SIT. We speculate that patients with AR cannot be isolated from house dust mite antigen, which may result in the continued existence of allergic humoral immunity and continuous generation of sIgE during early SIT. Consequently, the improvement of nasal symptoms in the early stage of SIT is likely to benefit from the cellular immunity mediated by T cells.

CD4^+^CD25^+^Treg was first discovered by Japanese scholar Sakaguchi in 1995 and was demonstrated to act as a vital immune modulator to maintain body’s immune homeostasis [16]. Studies have shown that CD4^+^CD25^+^Treg can inhibit excessive type I hypersensitivity mainly through its related cytokines IL-10 and TGF-β. Forkhead box p3 (Foxp3), a specific transcription factor of Treg, plays an essential role in Treg’s development and function. The mRNA expression level of Foxp3 can directly reflect Treg’s quantity and functional status in vivo [17]. It has been verified that Foxp3^+^Treg quantity and/or function decrease in the peripheral blood of patients with respiratory allergy, and recover after immunotherapy [2,18]. This study showed that Foxp3 mRNA expression level in PBMCs was significantly lower in the AR patients group than in the control group, suggesting a reduction of Treg quantity thus a hindered immunosuppressive function in AR. In contrast, in AR patients after SIT for one year, Foxp3 mRNA expression level significantly increased, suggesting the rectification of Treg quantity and/or function. In addition, with Foxp3 mRNA expression level ascending and VAS score descending, a significant negative correlation between them was maintained before and after SIT. These results suggest that Treg may play a pivotal role in the inhibition of patients’ symptoms caused by allergic inflammation.

The γδ T cells mainly distribute in the skin, small intestine, lung, and reproductive organs, and engage in the mucosal immune processes. Respiratory mucosal immunological barrier imbalance contributes largely to the occurrence of AR and asthma, and the role of γδ T cells play in these allergic diseases has received more attention [19]. It was reported that γδ T cells presented dual-directional regulatory function under different conditions via secreting diverse cytokines, which regulated what functional subsets γδ T cells were divided into [20]. For instance, γδ T cells showed a Th1 effect by generating IL-2 and INF-γ when bacterial infection occurred inside cells; but showed a Th2 effect by stimulating B cells with IL-4 and IL-5 with extracellular parasite infection. Additionally, human γδ T cells can be divided into several subsets based on different combinations of Vγ and Vδ chains at the variable regions of the T cell receptors. Among them, VγI subsets consist of Vγ2, 3, 4, 5 and 8, VγII subsets contain Vγ9, and VγIII subsets include Vγ10 [21]. In animals, Vγ1^+^γδ T subsets could aggravate airway hyperresponsiveness (AHR), acting as pro-inflammatory subsets. In contrast, Vγ4^+^γδ T subsets restrained AHR and had a protective effect on airway mucosa, belonging to anti-inflammation subsets [3,6]. Though there are differences in the distribution of γδ T cell subsets between humans and animals and between different organs, it is affirmed that γδ T cells contain both pro- and anti-inflammation subsets. The aim of this study was to determine the functional subsets that each VγI ~ III subfamilies belong to by testing the gene expression levels of VγI ~ III in PBMCs from AR patients before and after SIT. Our results showed that VγI ~ III expression levels in PBMCs from AR patients before SIT were all significantly lower than those in healthy controls, and VγIII expression level was negatively correlated to VAS score. After SIT for one year, changes in VγI and VγII expression levels in PBMCs had no statistical significance compared with those before SIT, while VγIII expression level significantly increased and was negatively correlated with VAS score. These results suggest that Vγ subfamilies, especially VγIII, were closely related to the occurrence of AR. In addition, VγIII subfamily might play an anti-inflammation role in the early stage of SIT, thereby alleviating clinical symptoms of AR patients.

In some immunological diseases, γδ T cells were found to influence the differentiation and function of Treg via secreting plenty of INF-γ, IL-17 or other cytokines for local inflammation [6,22]. Nevertheless, it has not yet been reported which subfamilies of γδ T cells are related to Treg in AR and SIT. This study showed that the expression levels of both VγI and VγII subfamily genes in PBMCs were positively related to that of Foxp3 gene, and the expression level of VγIII subfamily gene was positively related to that of Foxp3 gene both before and after SIT. These results strongly suggest that there exists a mutual regulatory effect and some dynamic equilibrium between Treg and TCR Vγ subfamilies, especially VγIII, in AR and the early stage of SIT. Future studies with larger sample size will further confirm the dynamic equilibrium between Treg and γδ T cells in peripheral blood as well as in nasal mucosa of AR patients before and after SIT.

## Conclusions

We characterized the expression pattern of Foxp3 gene and three TCR Vγ subfamily genes as well as the potential correlation between them in the PBMCs of AR patients before and after one-year-long effective SIT. The results showed that Treg and Vγ subfamily T cells were in a dynamic equilibrium in AR patients before and after effective immunotherapy for one year. The early improvement of symptoms following immunotherapy might be independent of the serum sIgE content in AR patients, but associated with the reconstitution of T cell immunity.

## Competing interests

The authors declare that they have no competing interests.

## Authors’ contributions

RZ and XLW coordinated the study, performed the real-time PCR and helped draft the manuscript; XKH and YLC prepared RNA and performed the serum test; YQL and GHZ helped to analyze data; QTY contributed to the concept development and study design. All authors read and approved the final manuscript.
